# Editorial: The role of microorganisms in the development and progression of cancer

**DOI:** 10.3389/fcimb.2023.1153372

**Published:** 2023-03-24

**Authors:** Maximilian Weniger

**Affiliations:** Department of General, Visceral and Transplantation Surgery, Ludwig-Maximilians-University, Munich, Germany

**Keywords:** cancer, microbiome, lung cancer, colorectal cancer, cervical cancer

The human body is home to trillions of microorganisms, collectively known as microbiota, which play a crucial role in maintaining health and well-being. Recent research has revealed that the gut microbiome in particular can significantly affect the development and progression of cancer. In this edition, we will present state-of-the art articles on the current understanding of the link between microbiota and cancer, with a specific focus on lung and colorectal cancer.

Lung cancer is the leading cause of cancer death worldwide, and non-small-cell lung cancer (NSCLC) is the most common form of the disease. Recent studies have found that the gut microbiome is altered in lung cancer patients, with specific changes in microbial composition and function.

The two studies included in this edition investigate the important links between lung cancer and the gut microbiome, which is altered significantly in lung cancer patients compared to healthy individuals (Chen et al.). Interestingly, gut microbiota and serum metabolic profiles have been closely related, providing new biomarkers for the diagnosis of early-stage NSCLC (Ni et al.).

Colorectal cancer (CRC) is one of the most common forms of cancer worldwide, and the gut microbiome is known to play a vital role in its etiology. The articles published in this edition analyze specific functions of the microbiome in CRC and their effect on CRC-related miRNA production, as well as the role of several bacteria including Fusobacterium nucleatum, Escherichia coli, Bacteroides fragilis, and Faecalibacterium prausnitzii (Xing et al.). Importantly, Fusobacterium nucleatum (Fn) seems to play a critical role in the development of CRC (Ou et al.). As discussed, one might hypothesize that prevention and treatment based on the relationship between Fn and CRC might be possible. Additionally, not only does the composition of the microbiome seem to play a critical role, but also metabolites produced by the intestinal microbiota influence colorectal cancer. Specifically, sodium butyrate can positively affect the immune system, intestinal barrier, anti-cancer treatment efficiency, and reduce mucositis induced by chemotherapy, making it a promising option for colorectal cancer patients (Kaźmierczak-Siedlecka et al.). Similarly, fecal metabolites not only seem to play a role in colorectal cancer, but they might also facilitate the diagnosis of gastritis. Interestingly, heptadecanoic acid and pentadecanoic acid in crosstalk with gut microbiota Erysipelotrichaceae_UCG-003 and Haemophilus correlate with chronic atrophic gastritis and could serve as novel biomarkers in the future (Gai et al.).

Furthermore, research on the microbiome has not only gained a foothold in gastrointestinal and pulmonary oncology, but also in studies on breast and cervical cancer. In this regard, a review included in this special issue illustrates the unique microbial composition in breast tissue and tumors, which could help develop novel therapeutic drugs (Song et al.).

Cervical cancer is a disease caused by the abnormal growth of cells in the cervix and is probably the best example of how the microbiome affects tumor biology. The well-established link between the human papilloma virus, cervical cancer, and the now-available vaccine is an excellent example of how microbiome research can lead to changes in real-world tumor therapy and outcomes (https://www.ncbi.nlm.nih.gov/pubmed/30638582). In this edition, an article investigates how HPV screening can detect cervical cancer (Zhang et al.). In addition, the impact of male HPV infection on both male and female HPV-associated cancers must not be overlooked (Zou et al.).

This edition of Frontiers in Cellular and Infection Microbiology serves to underline the importance of the microbiome in cancer and seeks to help increase our knowledge about this pivotal topic.

## Author contributions

The author confirms being the sole contributor of this work and has approved it for publication.

